# *TamiR159* Directed Wheat *TaGAMYB* Cleavage and Its Involvement in Anther Development and Heat Response

**DOI:** 10.1371/journal.pone.0048445

**Published:** 2012-11-01

**Authors:** Yu Wang, Fenglong Sun, Hua Cao, Huiru Peng, Zhongfu Ni, Qixin Sun, Yingyin Yao

**Affiliations:** State Key Laboratory for Agrobiotechnology, Key Laboratory of Crop Heterosis, Utilization (MOE) and Key Laboratory of Crop Genomics and Genetic Improvement (MOA), Beijing Key Laboratory of Crop Genetic Improvement, China Agricultural University, Beijing, People’s Republic of China; University of Georgia, United States of America

## Abstract

In Arabidopsis and rice, miR159-regulated *GAMYB-like* family transcription factors function in flower development and gibberellin (GA) signaling in cereal aleurone cells. In this study, the involvement of miR159 in the regulation of its putative target *TaGAMYB* and its relationship to wheat development were investigated. First, we demonstrated that cleavage of *TaGAMYB1* and *TaGAMYB2* was directed by miR159 using 5′-RACE and a transient expression system. Second, we overexpressed *TamiR159*, *TaGAMYB1* and *mTaGAMYB1* (impaired in the miR159 binding site) in transgenic rice, revealing that the accumulation in rice of mature miR159 derived from the precursor of wheat resulted in delayed heading time and male sterility. In addition, the number of tillers and primary branches in rice overexpressing *mTaGAMYB1* increased relative to the wild type. Our previous study reported that *TamiR159* was downregulated after two hours of heat stress treatment in wheat (*Triticum aestivum* L.). Most notably, the *TamiR159* overexpression rice lines were more sensitive to heat stress relative to the wild type, indicating that the downregulation of *TamiR159* in wheat after heat stress might participate in a heat stress-related signaling pathway, in turn contributing to heat stress tolerance.

## Introduction

MicroRNAs (miRNAs) are a class of small RNAs that serve as posttranscriptional negative regulators of gene expression in plants and animals [1], [2], [3], [4], [5], [6]. There are several indications that miRNAs regulate important aspects of plant development [6]. First, loss of function of genes encoding enzymes involved in miRNA biogenesis, such as *DCL1*, *HYL1*, *HEN1* and *AGO1*, results in developmental defects [7], [8], [9], [10]. Second, many target genes regulated by plant miRNAs encode putative transcription factors, that are involved in various developmental processes [11], including developmental transitions [12], [13], leaf growth [14], organ polarity [15], auxin signaling [16] and RNA metabolism [10], [17], [18]. For example, miR159-regulated *GAMYB-like* family transcription factors function in flower development and gibberellin (GA) signaling in cereal aleurone cells.

MiR159 is a conserved miRNA found in the dicots and monocots, and negatively regulates the expression of *GAMYB* genes at the posttranscriptional level [19]. In Arabidopsis, seven *GAMYB*-*like* genes share a conserved putative miR159 binding site. Among these genes, miR159-guided cleavage of *AtMYB33* and *AtMYB65* was detected using 5′-RACE and transient expression in *Nicotiana benthamiana* [20]. In cereals and Arabidopsis, *GAMYB* genes are predominantly expressed in the anthers and seeds, where miR159 is less accumulated [21], [22], this negative correlation in expression pattern provides evidence for miR159-directed *GAMYB* regulation. Overexpression of miR159 in rice results in decreased levels of *OsGAMYB* during inflorescence and flower malformation [21]. *AtMYB33* is transcribed broadly in *mAtMYB33* transgenic lines carrying an miR159-resistant binding site under the control of its endogenous promoter, indicating that miR159 restricts *AtMYB33* in specific tissues in wild-type plants [22]. Together, these results indicate that miR159 is part of a homeostatic mechanism to direct *GAMYB* transcript degradation in plants.

The target gene of miR159, *GAMYB*, was first identified as a downstream GA signaling target in aleurone cells of barley (*Hordeum vulgare* L.) [23]. During seed germination, *GAMYB* is activated by GA to promote the expression of genes encoding hydrolytic enzymes, including α-amylase, EII (1–3,1–4)-β-glucanase and cathepsin B-like protease, by binding to the cis-element GARE in their promoter regions [24], [25], [26]. GAMYB plays an important role in stamen development, demonstrated by loss-of-function mutations of GAMYB in rice that result in male sterility due to the delayed degradation of tapetum cells in stamen as well as defects in the formation of exines and Ubisch bodies [21], [27]. Similar to rice, the Arabidopsis genome includes *AtMYB33* and *AtMYB65*, which appear phylogenetically related to cereal *GAMYB* genes. Double mutant *myb33myb65* was also male sterile due to the involvement of the programmed cell death process (PCD), during which hypertrophy of the tapetum crushes the microspores [22], [27], [28]. Transgenic Arabidopsis overexpressing miR159 show pleiotropic morphological defects, such as anther defects due to the de-regulation of *AtMYB33* and *AtMYB65*, including those related to the anthers, male sterility, delayed flowering, reduced apical dominance and small siliques, which are suppressed in the *mir159abmyb33myb65* quadruple mutant [20], [29].

Several recent studies have indicated that the miR159-*GAMYB* pathway might also be involved in the abiotic stress response. For example, Reyes and Chua [30] reported that ABA-induced accumulation of miR159 is a homeostatic mechanism to desensitize hormone signaling during seedling stress response, directing *AtMYB33* and *AtMYB101* transcript degradation [30]. In our previous study, we found that miR159 was downregulated in wheat seedling leaves after heat stress for 2 hrs and that the expression pattern of its putative target *TaGAMYB* was negatively related to the accumulation of miR159, suggesting that miR159 and its putative targets may be involved in response to heat stress [31]. However, the relationship between the altered accumulation of miR159 and expression of its target, *GAMYB* genes in abiotic stress response has yet to be elucidated.

Here, we identified two full-length *GAMYB* genes *TaGAMYB1* and *TaGAMYB2,* which were putatively regulated by miR159 in wheat and experimentally confirmed that *TamiR159* directs the cleavage of two *TaGAMYB* transcripts. Spatial and temporal expression analyses indicated homeolog-specific expression patterns of *TaGAMYB1*, and all of three homeologous genes were similarly responsive to heat stress. To further investigate the role of *TamiR159* and *TaGAMYB1*, transgenic rice lines overexpressing miR159, *TaGAMYB1,* and m*TaGAMYB1* (impaired in the miR159 binding site) were generated. While both lines overexpressing *TamiR159* and m*TaGAMYB1* display an increased number of tillers, lines overexpressing *TamiR159* also demonstrate male sterility. Notably, we found that both transgenic rice lines overexpressing *TamiR159* and the Arabidopsis *myb33myb65* double mutant were both heat sensitive.

## Results

### MiR159-directed Cleavage of *TaGAMYB1* and *TaGAMYB2*, Putative *HvGAMYB* Orthologs

**Figure 1:**
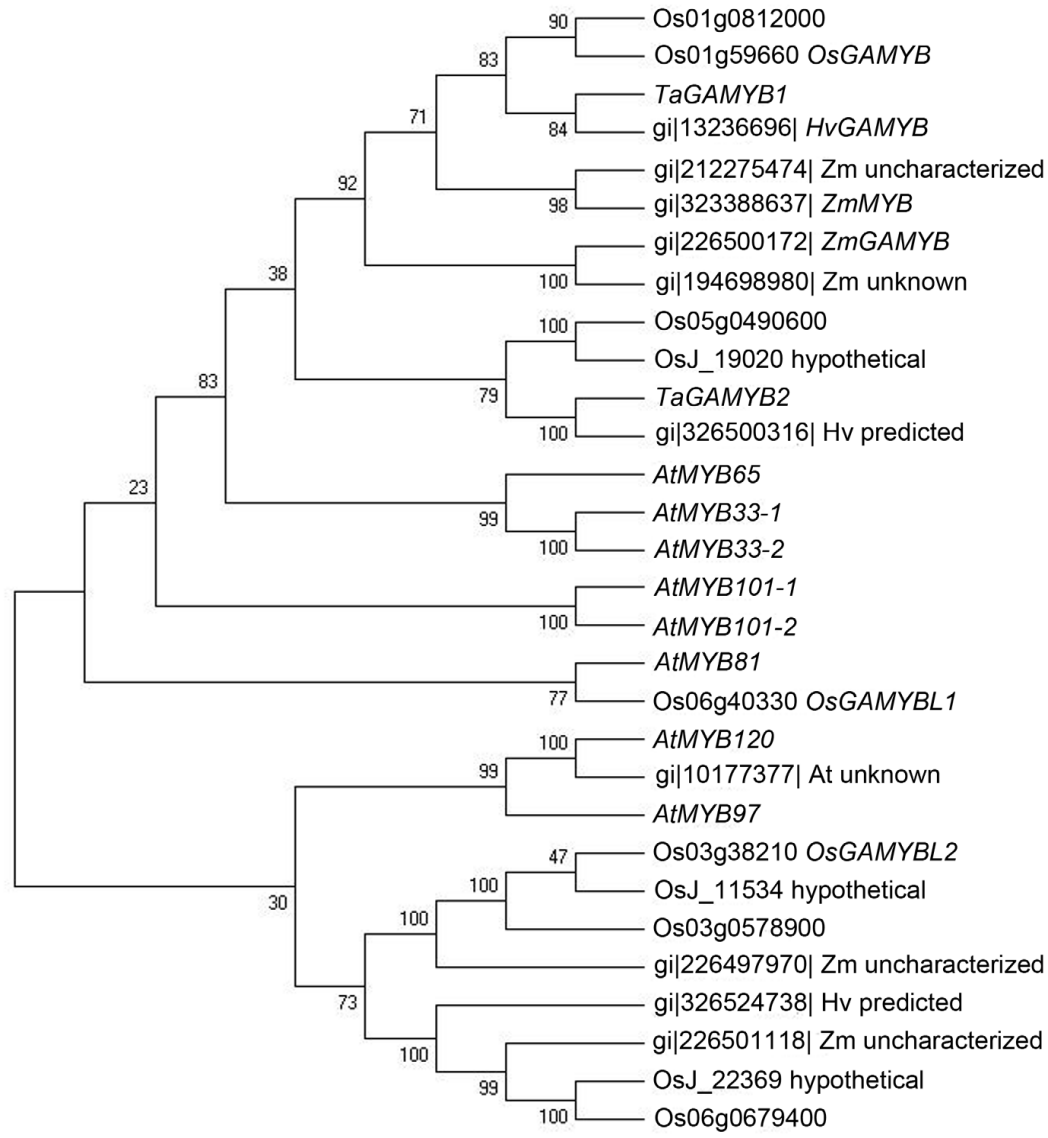
Phylogenetic tree of GAMYB proteins from *Triticum aestivum* (*Ta*), *Zea mays* (*Zm*), *Arabidopsis thaliana* (*At*), *Oryza sativa* (*Os*) and *Hordeum vulgare* (*Hv*). All GAMYB proteins were clustered using ClustalX, and the phylogenetic tree was generated by MEGA.

We previously reported two ESTs containing the reverse complementary binding site for miR159 [31]. Using *in silico* cloning, two unique sequences with full open reading frame were obtained and designated *TaGAMYB1* and *TaGAMYB2*. A sequence analysis revealed that these ORFs (open reading frames) were 1659 bp and 1383 bp respectively, and that each contained four canonical GAMYB domains (BOX1, BOX2, BOX3 and R2R3), which are highly conserved among rice, barley and *Arabidopsis* [32]. The phylogenetic tree of homologous GAMYB proteins showed that *TaGAMYB1*, *HvGAMYB* and *OsGAMYB* belong to one group due to their identical R2R3 and BOX2 domains. *TaGAMYB2* has less similarity at the R2R3 domain and was therefore classified to another group (Figure 1).

**Figure 2:**
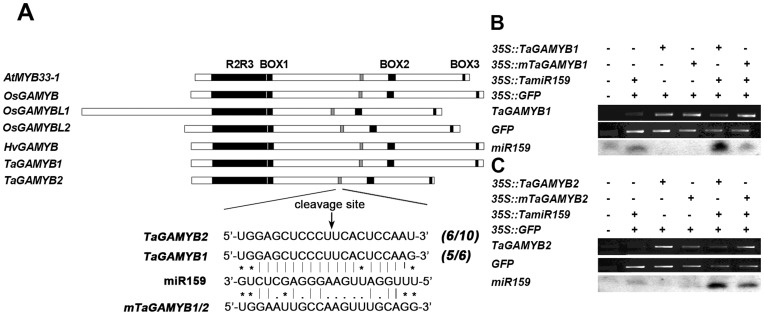
MiR159 directs the cleavage of *TaGAMYB* and the cleavage site. (A) 5′-RACE was performed to map the cleavage site of *TaGAMYB*. The conserved domains (R2R3, BOX1, BOX2 and BOX3) of GAMYB in three cereals and Arabidopsis are shown in black. The complementary sequences between miR159 and *GAMYB* genes are shown in grey. The arrow indicates the cleavage site, and the sequence of the mutated miR159 target site is illustrated (bottom). Numbers in italics indicate the proportion of clones analyzed that mapped to the miR159 cleavage position. (B) Expression analysis of miR159, *TaGAMYB1* and *mTaGAMYB1* in *N. benthamiana* leaves co-Agro-infiltrated with different combinations of *35S::TamiR159*, *35S::TaGAMYB1*, *35S::mTaGAMYB1* and *35S::GFP.* (C) Expression analysis of miR159, *TaGAMYB2* and *mTaGAMYB2* in *N. benthamiana* leaves co-Agro-infiltrated with different combinations of *35S::TamiR159*, *35S::TaGAMYB2*, *35S::mTaGAMYB2* and *35S::GFP.*

It was previously reported that miR159 negatively regulates *GAMYB* at the post-transcriptional level in both rice and Arabidopsis. We found reverse complementary binding sites for miR159 located between BOX2 and BOX3 domains in two *TaGAMYB* genes. To confirm *TaGAMYB* mRNA cleavage by miR159 *in vivo*, cleavage products were detected using a modified 5′-RACE procedure. The results demonstrated that 5 out of 10 clones showed the predominant cleavage site in *TaGAMYB1* at position 11 from the 5′ end of the miR159-*TaGAMYB1* complementary region. Similarly, 6 out of 10 clones of mapped the cleavage site in *TaGAMYB2* to the same nucleotide (Figure 2A). Our observations indicated that *TaGAMYB* mRNAs were cleaved at the miR159 complementary site in wheat leaves.

Next, to test whether *TamiR159* directs the cleavage of *TaGAMYB* mRNAs, we used an *Agrobacterium*-mediated delivery system to co-express *TamiR159* precursor and *TaGAMYB* target mRNA in *N. benthamiana* leaf tissue. For *TaGAMYB1*, four constructs (*35S::TamiR159*, *35S::TaGAMYB1*, *35S::mTaGAMYB1* and *35S::GFP*) were used for inoculation and were expressed in the leaf tissue. In tissues lacking the *35S::TamiR159* construct, basal levels of endogenous miR159 were hardly detected, while increased levels of miR159 were detected, indicating mature formation of miR159 in *N. benthamiana* leaves inoculated with the *35S::TamiR159* construct. In tissues inoculated with *35S::TaGAMYB1* construct, the *TaGAMYB1* transcript was detectable. Co-expression of *35S::TamiR159* with *35S::TaGAMYB1* led to obviously reduced mRNA levels of *TaGAMYB1* compared with the overexpression of *TaGAMYB1* alone. Furthermore, due to inability to bind with miR159, tissues co-expressing *35S::TamiR159* and *35S::mTaGAMYB1* (miR159 cleavage-resistant) did not exhibit obvious transcript changes relative to those transformed with the *35S::mTaGAMYB1* construct alone (Figure 2B). A similar procedure was performed using *TaGAMYB2,* the results of which suggested that the miR159 acts to cleave the two *TaGAMYB* transcripts efficiently (Figure 2C).

### Homoeolog-specific Expression Patterns of *TaGAMYB1*

**Figure 3:**
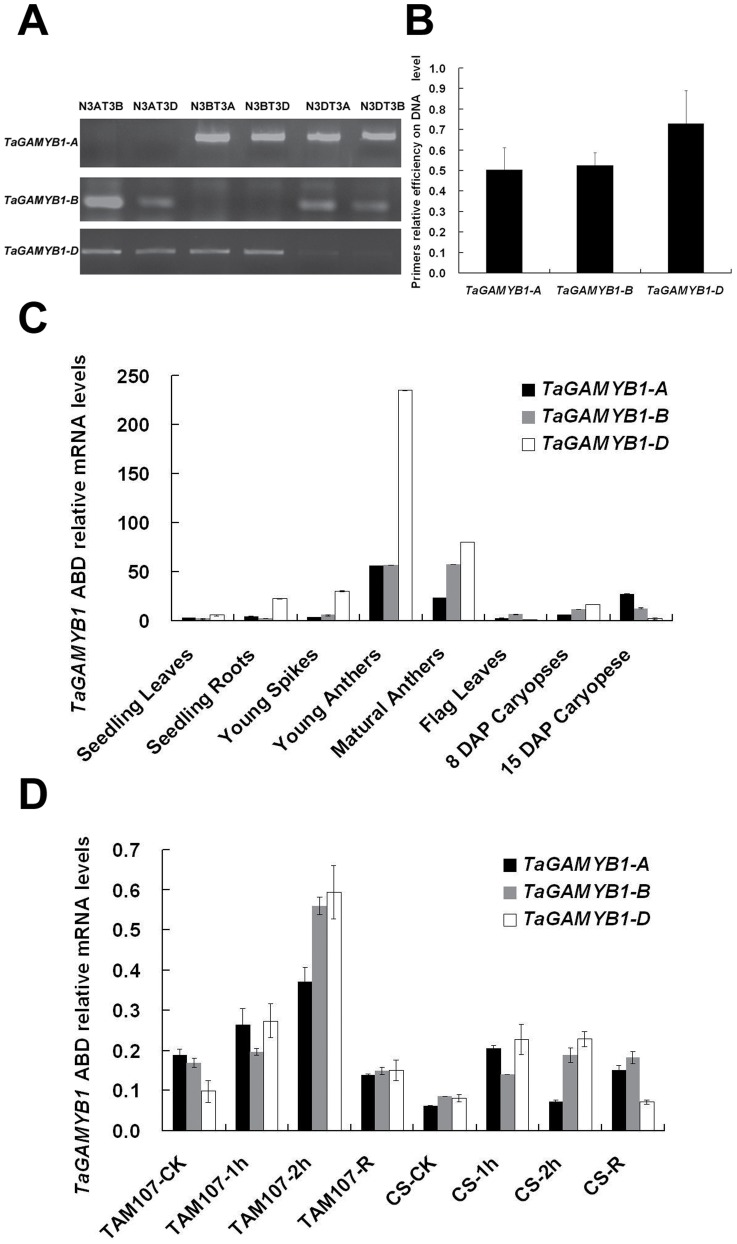
*TaGAMYB1-A, B, D* chromosome locations expression patterns in various tissues and responses to heat stress. (A) Genome-specific PCR amplification for the three homeologous *TaGAMYB1* genes. Each primer was used to amplify a Chinese Spring (CS) nulli-tetrasomic set. N3AT3B indicates a nulli-3A-tetra-3B line of CS, and so on. (B) Amplification efficiency of primers for the three homeologous *TaGAMYB1* genes estimated by Q-PCR using CS DNA. (C) The expression patterns of *TaGAMYB1-A, TaGAMYB1-B,* and *TaGAMYB1-D* in leaves, roots, young spikes, anthers and developing seeds. (D) The expression pattern of *TaGAMYB1-A, TaGAMYB1-B,* and *TaGAMYB1-D* in response to heat stress. Heat-tolerant cultivar TAM107 and heat-susceptible cultivar CS seedlings were treated at 42°C for 0.5 hr, 1 hr and 2 hrs. Those seedlings treated for 2 hrs were returned to normal growth conditions for 24 hrs (R).

Wheat is a hexaploid species that originated from three diploid ancestral species (*Triticum urartu*, *Aegilops speltoides* and *Aegilops tauschii*), thus providing the genome constitution of AABBDD [33]. To distinguish the expression of homeologous copies of *TaGAMYB1* from the A, B and D genomes, we designed specific primers to amplify the homeologous genes based on their sequence diversity in the 5′ untranslated region (5′UTR). A set of Chinese Spring nulli-tetrasomic lines were used to map homeologous genes on chromosomes 3A, 3B and 3D, we named these genes *TaGAMYB1-A, TaGAMYB1-B* and *TaGAMYB1-D*, respectively (Figure 3A).

The spatial and temporal expression patterns of the three homeologous *TaGAMYB1* genes in leaves, roots, young spikes, flag leaves, anthers and developing caryopses were determined by real-time PCR. *TaGAMYB1-A* and *TaGAMYB1-B* were highly expressed in both young and mature anthers, while *TaGAMYB1-D* was mainly expressed in young anthers. In addition, *TaGAMYB1-A* was highly expressed in seeds 15 days after pollination (DAP). Further comparison revealed significant differences in the relative mRNA abundance of the three *TaGAMYB1* homeologs, among which levels of *TaGAMYB1-D* mRNA were clearly higher than that of *TaGAMYB1-A* and *TaGAMYB1-B* (Figure 3C). To account for any differences in primer amplification efficiency, we also amplified *TaGAMYB1-A*, *TaGAMYB1-B* and *TaGAMYB1-D* using DNA templates, which revealed similar amplification efficiencies between the three templates (Figure 3B).

The expression patterns of the three homeologous genes *TaGAMYB1-A*, *TaGAMYB1-B*, and *TaGAMYB1-D* in response to high temperatures were also examined during a time course experiment using the heat-sensitive cultivar Chinese Spring and heat-tolerant cultivar TAM107. We observed that the expression of all three homeologous genes increased more than 2-fold after 2 hrs of heat treatment and then returned to normal expression levels after the plants recovered (Figure 3D). Furthermore, the expression profiles of the three *TaGAMYB1* homeologous genes of the heat-tolerant and heat-sensitive cultivars were compared. These results demonstrated that after heat treatment, the level of *TaGAMYB1* mRNA alteration in TAM107 was much higher than in CS, indicating that the expression of *TaGAMYB1* genes is more inducible in the heat-tolerant cultivar TAM107.

### Overexpression of miR159 and *TaGAMYB1* in Rice Leads to an Abnormal Phenotype

**Figure 4:**
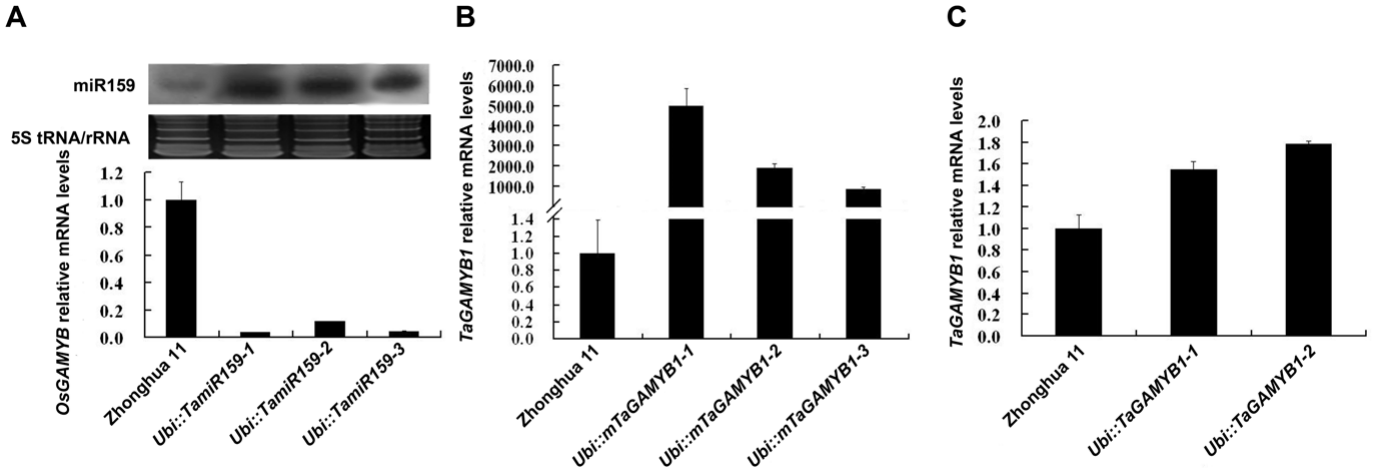
Expression of *TamiR159*, *TaGAMYB1* and *OsGAMYB* in transgenic *Ubi::TamiR159*, *Ubi::TaGAMYB1* and *Ubi::mTaGAMYB1* rice lines. (A) Real-time PCR and Northern blot analysis of leaves from *Ubi::TamiR159* transgenic plants were used to determine the relative expression of endogenous *OsGAMYB* and mature miR159 levels. (B) Real-time PCR was used to determine the relative expression of *TaGAMYB1* in leaves from *Ubi::TaGAMYB1* transgenic plants. (C) Real-time PCR was used to determine the relative expression of *TaGAMYB1* in leaves from *Ubi::mTaGAMYB1* trangenic plants.

To further elucidate the biological function of *TamiR159* and its target *TaGAMYB1*, we generated transgenic rice that overexpresses the wheat miR159 precursor, *TaGAMYB1* and *mTaGAMYB1* (with a miR159 cleavage-resistant site) under the control of the strong and constitutive ubiquitin promoter. The transgenic lines were designated *Ubi::TamiR159*, *Ubi::TaGAMYB1* and *Ubi::mTaGAMYB1*, respectively, and the expression levels of miR159, *TaGAMYB1* and endogenous *OsGAMYB* were measured (Figure 4). In *Ubi::TamiR159* lines, mature miR159 was highly expressed, and *OsGAMYB* transcripts were almost undetectable, suggesting that miR159 derived from a wheat precursor has the capability to direct cleavage of the rice endogenous *OsGAMYB in* *vivo* (Figure 4A). In *Ubi::TaGAMYB1* lines, *TaGAMYB1* transcripts increased less than 2-fold due to the cleavage of endogenous rice miR159 (Figure 4B). In *Ubi::mTaGAMYB1* lines, *TaGAMYB1* transcripts dramatically increased more than 1000-folds (Figure 4C).

**Figure 5:**
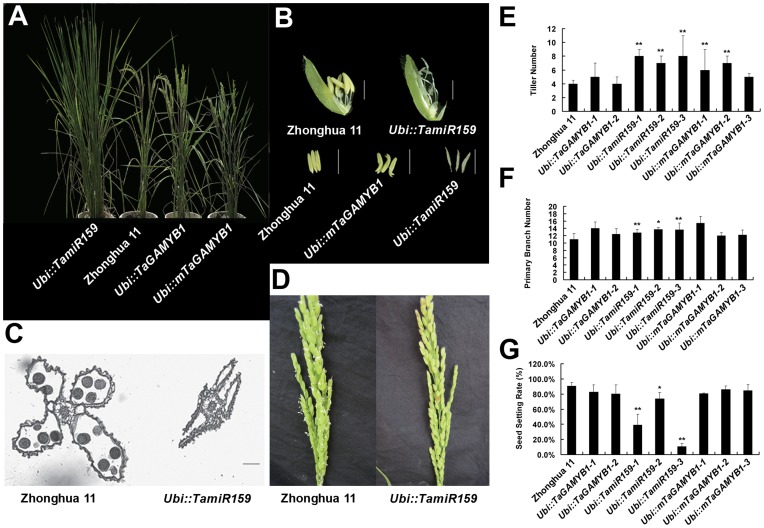
Phenotypes of Zhonghua11 and transgenic *Ubi::TamiR159*, *Ubi::TaGAMYB1* and *Ubi::mTaGAMYB1* rice under normal field conditions. (A) *Ubi::TamiR159-1*, Zhonghua11, *Ubi::TaGAMYB1* and *Ubi::mTaGAMYB1–2* lines at heading time. (B) Flowers and stamens of Zhonghua11, *Ubi::TamiR159-1* and *Ubi::mTaGAMYB1–2* lines. Bars = 2 mm. (C) Transverse sections of Zhonghua11 and *Ubi::TamiR159-1* plant anthers at the mature pollen stage. Bar = 20 µm. (D) Panicles of Zhonghua11 and *Ubi::TamiR159-1* plants at heading time. (E) Tiller number of Zhonghua11, *Ubi::TamiR159*, *Ubi::TaGAMYB1* and *Ubi::mTaGAMYB1* plants. (F) Number of primary branches of Zhonghua11, *Ubi::TamiR159*, *Ubi::TaGAMYB1* and *Ubi::mTaGAMYB1* plants. (G) Seed setting rate of Zhonghua11, *Ubi::TamiR159*, *Ubi:TaGAMYB1* and *Ubi::mTaGAMYB1* plants. **: P<0.01, *: P<0.05.

First, *Ubi::TamiR159* transgenic lines and wild-type Zhonghua11 were phenotyped after the tillering stage and were observed to detect any obvious phenotypic changes. *Ubi::miR159* plants showed dramatic morphological changes, including a significantly (P<0.05) increased number of tillers (two times more than Zhonghua11) and delayed flowering (20 days delay) (Figure 5A, E). *Ubi::TamiR159* plants developed a 10% increased in the number of primary branches of panicles when compared to Zhonghua11 (Figure 5F), but the number of secondary branches was unaffected. The anthers of *Ubi::TamiR159* plants were shrunken with no pollen in the anther sac and were incapable of emerging from the glume (Figure 5B, C, D), leading to a reduction in fertility as indicated by a 30%–80% reduction in seed setting rate (Figure 5G). Other flower structures, such as the anther walls and connectivum, maintained a normal morphology (Figure 5C).

Second, the phenotype of *Ubi::mTaGAMYB1* transgenic lines was also compared with that of wild-type Zhonghua11. Except for increased tiller numbers and delayed heading time, *Ubi::mTaGAMYB1* lines developed normally, including the number of secondary branches, seed setting rate, 1000-gain weight and the length of the main panicle. In addition, the vegetative and heading stages of *Ubi::TaGAMYB1* were similar to those of Zhonghua11.

### MiR159-*GAMYB* Pathway Might Contribute to Heat Stress Response

**Figure 6:**
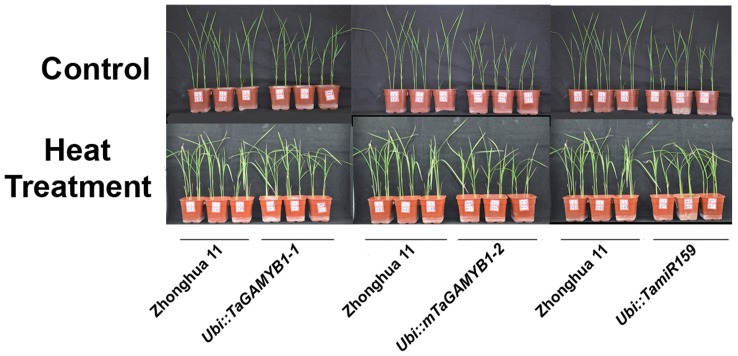
Heat tolerance testing of *Ubi::TamiR159*, *Ubi::TaGAMYB1* and *Ubi::mTaGAMYB1* transgenic seedlings. The top panel shows seedlings before heat treatment. The bottom panel shows seedlings recovered at 45°C/42°C for 5 days for 5 days after heat treatment.

Considering that miR159 and *TaGAMYB1* mRNA are heat-inducible, we examined the performance of plants overexpressing miR159 and *TaGAMYB1* under heat stress when compared to the wild type. Transgenic lines were grown under normal conditions for 2 weeks and then treated with 45°C/42°C (day/night) for 5 days. Seedlings were then allowed to recover for 5 days under normal conditions. After heat treatment, transgenic *Ubi::miR159* plants grew slower and withered more when compared to Zhonghua11 after heat treatment. In addition, *Ubi::mTaGAMYB1* plants did not show any obvious differences following heat stress (Figure 6). These results suggest that miR159-directed cleavage of *OsGAMYB* may participate in the heat stress response in *Ubi::TamiR159*.

**Figure 7:**
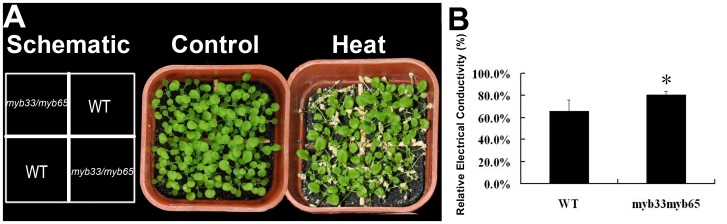
Heat tolerance testing of *Arabidopsis* wild-type (WT) and *myb33myb65* double mutant plants. (A) Phenotype of WT and *myb33myb65* 2-weeks-old seedlings after heat stress for 4 hr at 44°C Identical samples was planted diagonally. WT seedlings were planted in the northwest and southeast corners, while double mutant seedlings were planted in the other two corners as indicated in the schematic. (B) Relative electrical conductivity test of WT and *myb33myb65* double mutants after heat treatment.

*GAMYB* genes have a conserved biological function in anther development, promoting us to investigate whether homologous *GAMYB* genes in Arabidopsis have an effect on heat tolerance. In Arabidopsis, *GAMYB*-like genes *AtMYB33* and *AtMYB65* were found to be functionally redundant [22]. Therefore, we measured heat tolerance in *myb33myb65* double mutants. Arabidopsis *myb33myb65* double mutant seedlings grew slower and weaker after being exposed to 44°C for 4 hr, indicating that Arabidopsis *myb33myb65* double mutants, similar to *Ubi::miR159* transgenic rice, are heat sensitive (Figure 7A). Next, we determined the relative electrical conductivity as an index of membrane injury, which was higher in *myb33myb65* double mutants when compared to wild-type (Figure 7B). These results demonstrate that a loss of function of *GAMYB* genes affects heat tolerance, further implicating a potential role for *GAMYB* genes in the heat stress response.

## Discussion

In this study, we identified two full-length *GAMYB* genes putatively regulated by miR159 in wheat and confirmed using 5′-RACE and a transient expression system that TamiR159 directs the cleavage of two *TaGAMYB* transcripts. Moreover, we generated rice transgenic lines with wheat miR159 precursor overexpression, in which an increase mature miR159 and decrease in endogenous *OsGAMYB* were detected. In addition, miR159 and *TaGAMYB1* expression levels were negatively correlated. For example, miR159 was almost absent in spikes at the booting stage and in developing endosperms in wheat [21], [34], whereas *TaGAMYB* showed high expression levels in anthers and seeds, similar to previous reports in Arabidopsis and rice [21], [22]. Taken together, our data provided further evidence for the biogenesis of miR159 and its cleavage of target genes being conserved among different plant species.

Although all three homeologs on A, B and D genome for the majority of genes were assumed to be expressed in hexaploid wheat, it is of great interest to reveal how this expression of is regulated [35]. Our data demonstrate distinct spatio-temporal expression patterns between the three homeologus *TaGAMYB1* genes. For example, *TaGAMYB1-A* and *TaGAMYB1-B* were highly expressed in both young and mature anthers, whereas *TaGAMYB1-D* was mainly expressed in young anthers. *TaGAMYB1-A* was also highly expressed in seeds 15 days after pollination (DAP). There was no sequence variation in the miR159 binding site among these three homeologous genes, meaning that they shared the same miR159-directed cleavage machinery. Although the abundance of miR159 is below detection levels in anthers and seeds, the unequal expression levels of these three homeologous genes suggest that the involvement of either a homeologous-specific cis-element in the promoter region or epigenetic regulation. In support of the latter hypothesis, it has been reported that *WLHS1-B* and *WLHS1-D* have a complete MADS box gene structure, whereas *WLHS1-B* is predominantly silenced by cytosine methylation [35].

In the rice *gamyb* mutant and Arabidopsis *myb33myb65* double mutants, male sterility results from the defective PCD in the tapetum layer and eventual failure of the anther wall by collapse. Similar to *gamyb* and *myb33myb65* mutants, *Ubi::TamiR159* transgenic lines, in which rice *OsGAMYB* was silenced, showed male sterility. However, in *Ubi::TamiR159* plants at the mature pollen stage, the empty locule (without pollen grain) was enclosed by a structurally normal anther wall rather than filled with expanded tapetal cells as in the rice *gamyb* mutant [27]. These observations suggest that tapetum layers break down normally in *Ubi::TamiR159* plants and that the male sterility occurs due to aberrant microsporogenesis or developmental blockage that leads to a lack fo pollen production. Collectively, we speculate that the underlying male sterility mechanism triggered by wheat miR159 is different from that of rice and Arabidopsis. One possible explanation is that other putative targets of miR159 might also contribute to the failure of pollen production.

In addition to defective anther development, the delayed heading time of both *Ubi::TamiR159* and *Ubi::mTaGAMYB1* transgenic plants is consistent with Arabidopsis studies in which miR159 or *mAtMYB33* (miRNA binding site-disrupted) overexpression results in late flowering. Moreover, the increased number of tillers in *Ubi::TamiR159* and *Ubi::mTaGAMYB1* plants might be a derivative of developmental retardation due to prolongation of the vegetative stage. This common phenotype suggests that the miR159-*GAMYB* system is critical for the transition from vegetative stage to reproductive stage and is conserved between monocots and dicots. However, there was non-conformity between Arabidopsis plants overexpressing m*AtMYB33* and transgenic *Ubi::mTaGAMYB1* plants. In Arabidopsis, overexpression of *AtMYB33* had pleiotropic effects on morphogenesis due to its broad action throughout the whole plant, whereas, despite global expression of *mTaGAMYB*, *Ubi::mTaGAMYB1* plants developed normally except for delayed heading time and increased number of primary branches. One possible reason for this observation is that *GAMYB* function in monocots and dicots diverged during evolution by mediating the expression of different sets of downstream genes, as *AtMYB33* and *TaGAMYB1* are in different clades (Figure 1). There were also reported differences between reported rice miR159 overexpression lines and *Ubi::TamiR159* transgenic lines. The length of the first internode of miR159 overexpression plants and *gamyb* mutants was shorter than that of wild type [21], but *Ubi::TamiR159* plants did not exhibit this difference, nor did they differ in plant height compared with wild type. We speculated that genotypic background impacts gene function.

High temperature, often combined with drought stress, causes yield loss and reduces the grain quality in wheat. We previously reported a diverse set of wheat miRNAs responsive to heat stress, among which miR159 was downregulated after high temperature treatment for 2 hrs [31]. In this study, we found three homeologous genes of the miR159 to be upregulated after high temperature treatment for 2 hrs, this upregulation was more dramatically induced in the heat-tolerant variety than in the heat-sensitive one. Due to the negative correlation between miR159 accumulation and *TaGAMYB1* expression during heat stress, we further tested the heat tolerance of transgenic rice containing *Ubi::TamiR159, Ubi::TaGAMYB1* and *Ubi::mTaGAMYB1* constructs. The results revealed that *Ubi::TamiR159* transgenic lines were heat sensitive, indicating that overexpression of miR159, combined with downregulation of *GAMYB* expression leads to a loss of heat tolerance. Interestingly, we also found that *TaGAMYB1* homologous genes *AtMYB33* and *AtMYB65* in Arabidopsis were also heat-inducible (data not shown). Moreover, the heat tolerance of the Arabidopsis *myb33myb65* double mutant was also reduced. In addition, GAMYB acted as a regulator of α-amylase by binding to the GARE element in aleurone cells. It has also been reported that three α-amylase genes in Arabidopsis are induced by heat stress, consequently increasing the soluble sugar content [36], [37], [38]. Given our data, we propose that the alteration of *TamiR159-TaGAMYB1* interaction might be involved in the heat stress response through the *GAMYB*-amylase pathway for starch degradation. It should be noted that overexpression of *GAMYB* did not improve heat tolerance at the seedling stage. Therefore, the causal relationship between *TamiR159-TaGAMYB1* interaction and heat stress tolerance requires further investigation.

## Materials and Methods

### Transformation Vectors and Generation of Transgenic Plants

The full-length cDNA of *TaGAMYB* was obtained from Chinese Spring anthers and confirmed by sequencing with SP6 and T7 primers. The sequence-confirmed clone containing the full-length cDNA of *TaGAMYB1* was digested by *Spe* I and *Kpn* I and cloned into the binary expression vector *pCAMBIA1301U* (driven by a maize *ubiquitin* promoter, *Ubi::TaGAMYB1*). An miR159 binding site mutant version of the *TaGAMYB1* transgene (*Ubi::mTaGAMYB1*) was generated by PCR using mutated *mTaGAMYB1* primers, this fragment was inserted to *pCAMBIA1301U* after digestion with *Spe* I and *Kpn* I. The precursor of miR159 in wheat was amplified and from genomic DNA by PCR using specific primers listed in SI Table1 digested with *BamH* I and *Kpn* I and cloned into *pCAMBIA1301U* (*Ubi::TamiR159*). Each of these constructs was introduced into Zhonghua11 (*Oryza sativa L. ssp. japonica*) by *Agrobacterium*-mediated transformation. *TaGAMYB* and *mTaGAMYB* cDNA fragments were amplified with primers possessing *Xba* I and *Kpn* I sites and cloned into *pCAMBIASuper1300* (*35S::TaGAMYB*, *35S::mTaGAMYB*). The precursor of miR159 was also cloned into the *Sma* I */Sac* I sites of *pCAMBIASuper1300* (*35S::TamiR159*). All primers are listed in Table S1.

### Plant Materials and Heat Treatments

Two wheat genotypes, heat-susceptible ‘Chinese Spring’ (CS) and heat-tolerant ‘TAM107’ were used in this study. Seeds were surface-sterilized and kept in the culture room maintained at 22°C/18°C (day/night), 12 hr/12 hr (light/dark), and 60% relative humidity for 10 days. For expression analysis, seedlings were exposed to heat stress for 0.5 hr,1 hr and 2 hr at 40°C and 2 hrs-treated seedlings were then returned to normal conditions for 24 hrs. After heat treatments, seedling leaves were frozen immediately in liquid nitrogen and stored at −80°C for further use.

Seeds of the wild-type rice Zhonghua11 and transgenic lines were soaked in deionized water overnight at 30°C in the dark, transferred to pots containing soil and grown in a chamber at 28°C/25°C (day/night), 14 hr/10 hr (light/dark), and 60% relative humidity. At the 3-leaf stage, seedlings were subjected to heat stress at 45°C/42°C (day/night) for 5 days and transferred to normal conditions for 5 days to recover. All experiments were conducted with three biological replicates and were repeated at least twice. For phenotype determination, transgenic and wild-type rice were grown in the fields.

The seeds of Arabidopsis ecotype *Columbia* wild type and the double mutant *myb33myb65* were sown onto soil and stratified at 4°C in the dark before being grown in 22°C growth cabinets. The seedlings were treated for 4 hr at 42°C with 60% relative humidity and were transferred to normal conditions for 5 days to recover.

### Gene Expression Analysis

For Northern blot hybridization, total RNA was extracted from rice and Arabidopsis using the TRIzol™ reagent (*Invitrogen*, Carlsbad, CA, USA) according to the manufacturer’s instructions. Low molecular weight RNA was enriched by 0.5 M NaCl and 10% PEG-8000 precipitation. A total of 10 µg of low molecular weight RNA was loaded per lane, resolved on a denaturing 15% polyacrylamide gel, and transferred electrophoretically to Hybond-N+ membranes (Amersham Biosciences, Buckinghamshire, UK). Membranes were EDC crosslinked [39]. DNA oligonucleotides complementary to miR159 were end-labeled with γ-32P-ATP using T4 polynucleotide kinase (TaKaRa, Dalian, China). Membranes were prehybridized for more than 8 hrs and hybridized overnight using Church buffer at 37°C. Blots were washed three times (two times with 2× SSC +1% SDS and one time with 1× SSC +0.5% SDS) at 50°C. The membranes were briefly air dried and then exposed to X-ray films for autography at −80°C.

For real-time PCR analysis, total RNA was treated with DNase (*Promega*, Madison, USA) and reverse-transcribed using *M-MLV™* Reverse Transcriptase (*Promega*, Madison, USA) according to the manufacturer’s instructions. Real-time quantitative PCR was performed on an optical 96-well plate with the C1000™ Thermal Cycler CFX96™ Real-Time System (BIO-RAD) and ABI PRISM 7500 real-time PCR system using SYBR Premix Ex Taq™ (TaKaRa). The PCR thermal cycles were 95°C for 10 s and 40 cycles at 95°C for 5 s; 60°C for 10 s and 72°C for 20 s. Gene-specific primers were designed using DNAMAN software to quantify *TaGAMYB* alleles and *OsGAMYB* are listed in SI Table 1. For the *GAMYB* genes in rice, primers were designed to span the cleavage site. Three *TaGAMYB* genes primers sets were designed in the 5′UTR to distinguish homeologous alleles [40]. The expression of each gene was normalized to β-actin of each species (Table S1).

### 5′-RACE (Rapid Amplification of 5′ cDNA Ends)

5′-RACE was conducted according to the manufacturer’s instructions (*Invitrogen*). Briefly, total RNA was extracted from spikes of CS with TRIzol™ reagent (*Invitrogen*). Reverse transcription reactions were performed with adaptive primers. cDNA templates were amplified for two rounds of PCR with universal sense primers provided in the kit and two gene-specific primers (Table S1) designed by DNAMAN. Nested PCR products were cloned into the *pEASY* vector (*Promega*, Madison, USA) and sequenced.

### Infiltration of *Agrobacterium Tumefaciens* into *N. benthamiana*

*35S::TaGAMYB1 (35S::TaGAMYB2)*, *35S::mTaGAMYB1 (35S::mTaGAMYB2)*, *35S::TamiR159* and *35S::GFP* (provided by the Gong lab) were introduced into *Agrobacterium tumefaciens* strain GV3101, and the bacteria were injected into *N. benthamiana* leaves with a syringe. For co-injections of two or three different constructs, bacteria were resuspended in infiltration medium (0.5× Murashige and Skoog salts, 5% sucrose, 0.5 g/l MES) at OD600 = 1 and incubated for 3 hrs at room temperature with 150 µM acetosyringone. Zones of infiltration were harvested for RNA isolation 2 days post injection. The *35S::GFP* construct was used as a control for the co-Agro-infiltration.

### Measurement of Electrical Conductivity

Central parts of Arabidopsis leaves of the same size were collected using a paper punch. Ten pieces of leaves taken from wild-type and *myb33myb65* double mutant plants were submerged in a clean test tube containing 10 ml ddH_2_O. All tubes were stored at room temperature for 24 hrs and then submerged in a water bath at 40°C for 50 min. Electrical conductivity was measured with Eco Scan Series CON5™ conductivity/°C Meter (EUTECH instruments) according to the manufacturer’s instruction reading in µS/cm. All tubes were heated at 100°C for 10 min and cooled at room temperature for 24 hrs. Electrical conductivity was then measured again with reading in µS/cm. The final results were calculated based on the formula n/m*100%.

### Histological Analysis

Rice anthers were fixed in formalin:acetic acid:70% ethanol (1∶1:18) and dehydrated through a graded ethanol series. The tissues were then embedded in LR White resin and sliced into 2 µm sections. Sections were stained with hematoxylin and viewed under a Leica DFC420 CCD microscope.

## Supporting Information

Table S1 **Primers and probes used in this study.**
(DOC)
