# Supplementary material for: TamiR159 Directed Wheat TaGAMYB Cleavage and Its Involvement in Anther Development and Heat Response
Source: PLoS One. 2012 Nov 1;7(11):e48445. doi: 10.1371/journal.pone.0048445 (PMC3486836; doi:10.1371/journal.pone.0048445)
Supplement: Table S1 — Primers and probes used in this study. (DOC) [file pone.0048445.s001.doc]

**Supplementary Table 1** Primers and probes used in this study

| **Primers for 5'RACE** | **Sequence (5'-3')** |  |
| --- | --- | --- |
| 5’adapter primer | CGACTGGAGCACGAGGACACTGA |  |
| 5’nest primer | GGACACTGACATGGACTGAAGGAGTA |  |
| *TaGAMYB1*-outer | AGCTCTTCCAAGAGACCGCTGTT |  |
| *TaGAMYB1*-nest | GACTTCACTGACGGAGTTGCTGTC |  |
| *TaGAMYB2*-outer | AGAGCTTACTGCATGAGCTTCTGG |  |
| *TaGAMYB2*-nest | AACTGACCGCTGTTCCCTGGTAT |  |
| Sp6 | ATTTAGGTGACACTATA |  |
| T7 | TAATACGACTCACTATAGGG |  |
| **Primers for Transition Expression** | **Sequence (5'-3')-left** | **Sequence (5'-3')-right** |
| *TaGAMYB1*-35S | TGCTCTAGAATGTACCGGGTGAAGAGCGAGAG | CGGGGTACCTCATTTGAATTCCTCCGACAT |
| *TaGAMYB2*-35S | GCTCTAGAGCACCGTGTTTCTGAGAG | GGGGTACCACTGTAGACCCGTTCGAATC |
| mutation*TaGAMYB1*-35S | CAATGGTCCTTTGAAGATGGAATTGCCAAGTTTGCAGGATACC | GGTATCCTGCAAACTTGGCAATTCCATCTTCAAAGGACCATTG |
| mutation*TaGAMYB2*-35S | GATCCAGCAAACTGCAAACTTGGCAATTCCATCTTC | GAAGATGGAATTGCCAAGTTTGCAGTTTGCTGGATC |
| pre-miR159-35S | TCCCCCGGGGCACCGTGTTTCTGAGAG | CGAGCTCACTGTAGACCCGTTCGAATC |
| **Primers for Transformation Rice** |  |  |
| *TaGAMYB1*-Ubi | GGACTAGTATGTACCGGGTGAAGAGCGAGAG | GGGGTACCTCATTTGAATTCCTCCGACAT |
| mutationTaGAMYB1-Ubi | CAATGGTCCTTTGAAGATGGAATTGCCAAGTTTGCAGGATACC | GGTATCCTGCAAACTTGGCAATTCCATCTTCAAAGGACCATTG |
| pre-miR159-Ubi | CGGGATCCGCACCGTGTTTCTGAGAG | CGGGTACC ACTGTAGACCCGTTCGAATC |
| ***GAMYB* Q-PCR** | **Sequence (5'-3')-Left** | **Sequence (5'-3')-Right** |
| *TaGAMYB1*-A | TCCACCGAGACCCGCCGC | CCACCGGCGAGTCCATCT |
| *TaGAMYB1*-B | GAGTATGCTGCTCCCTTCAG | AGAAGAATGCGTCTGGCCT |
| *TaGAMYB1*-D | AGCCGGGAGGAGGTGCTG | TGTGAGGCGACCCTCCGCT |
| TaActin | GGAATCCATGAGACCTAC | GACCCAGACAACTCGCAAC |
| *OsGAMYB* | GCGCAGTTCTTCCAGTTCC | GAAGGGAGCTCGGCTCTG |
| OsActin | CATCTTGGCATCTCTCAGCAC | AACTTTGTCCACGCTAATGAA |
| *TaGAMYB1* | CCTGAATTGAGCGACACC | ACACACTCCGACTTCACTG |
| *TaGAMYB2* | GTGGGGCGATTTCATTGAT | TTGAGTATGCGGACCAGTTG |
| miR159 | CAGAGCTCCCTTCAATCCAAA |  |
